# Editorial Notes, News and Miscellany

**Published:** 1913-03

**Authors:** 


### Editorial Notes, News and Miscellany.

BEWARE OF A NOSTRUM called "Freckleater." It will tan the skin and make it look like a leather pocketbook.

HONORARY MEMBERSHIP in the New York Medico-Legal Society was recently conferred upon Justice Goff and Attorney Whitney, of New York, and Dr. F. E. Daniel, of Texas, "for eminent public services."

HON. CLARK BELL, founder of the New York Medico-Legal Society, and its President since 1872—forty years—and founder of the *Medico-Legal Journal* in 1883, announces that with the March number that publication will be discontinued.

THE NEW STATE BOARD OF MEDICAL EXAMINERS.—Drs. W. B. Collins, of Houston county; W. L. Crosthwaite, of McLennan county; Geo. L. Baber, of Wood county; J. H. Evans of Anderson county, and E. B. Osborn, of Johnson county, as representatives of the regular school of medicine; Drs. J. F. Bailey of McLennan county, and Paul M. Peck, of Bexar county, as representatives of the Osteopaths; Drs. M. E. Daniel, of Fannin county, and Geo. W. Johnson, of Bexar county, as representatives of the Eclectics; Drs. T. J. Crowe, of Dallas county, and H. C. Morrow, of Travis county, as representatives of the Homeopaths.

---

CUTTING OFF THE PUPPY'S TAIL.—A tenderhearted woman wanted her puppy's tail cut off, but thought it would hurt to cut it all off at once, so she cut off an inch each day. Four years ago the Legislature caused the heretofore all-night saloons to close at 12. The present Legislature ("platform demand") requires them to close at 9:30, thus cutting off an inch more of the evil; and it is thought that the next Legislature will cut off another inch and cause them to close at 6,—three whacks and three inches,—can't afford to cut it all off at once—"unconstitutional"; hurt too bad. The Legislature has also passed a law against the *pollution of streams,* but exempts all towns below 1500 population; couldn't go the whole puppy; hurt too bad.

